# Stress Memory in Seagrasses: First Insight Into the Effects of Thermal Priming and the Role of Epigenetic Modifications

**DOI:** 10.3389/fpls.2020.00494

**Published:** 2020-04-28

**Authors:** Hung Manh Nguyen, Mikael Kim, Peter J. Ralph, Lázaro Marín-Guirao, Mathieu Pernice, Gabriele Procaccini

**Affiliations:** ^1^Stazione Zoologica Anton Dohrn, Villa Comunale, Naples, Italy; ^2^Seagrass Ecology Group, Oceanographic Center of Murcia, Spanish Institute of Oceanography, Murcia, Spain; ^3^Climate Change Cluster (C3), University of Technology Sydney, Sydney, NSW, Australia

**Keywords:** seagrasses, thermal priming, gene expression, *Posidonia australis*, *Zostera muelleri*, epigenetic

## Abstract

While thermal priming and the relative role of epigenetic modifications have been widely studied in terrestrial plants, their roles remain unexplored in seagrasses so far. Here, we experimentally compared the ability of two different functional types of seagrass species, dominant in the Southern hemisphere, climax species *Posidonia australis* and pioneer species *Zostera muelleri*, to acquire thermal-stress memory to better survive successive stressful thermal events. To this end, a two-heatwave experimental design was conducted in a mesocosm setup. Findings across levels of biological organization including the molecular (gene expression), physiological (photosynthetic performances and pigments content) and organismal (growth) levels provided the first evidence of thermal priming in seagrasses. Non-preheated plants suffered a significant reduction in photosynthetic capacity, leaf growth and chlorophyll *a* content, while preheated plants were able to cope better with the recurrent stressful event. Gene expression results demonstrated significant regulation of methylation-related genes in response to thermal stress, suggesting that epigenetic modifications could play a central role in seagrass thermal stress memory. In addition, we revealed some interspecific differences in thermal responses between the two different functional types of seagrass species. These results provide the first insights into thermal priming and relative epigenetic modifications in seagrasses paving the way for more comprehensive forecasting and management of thermal stress in these marine foundation species in an era of rapid environmental change.

## Introduction

Plants, as sessile organisms, have developed sophisticated mechanisms to efficiently respond to environmental changes as they cannot quickly escape from potentially stressful conditions. Some of these mechanisms are included within the concept of stress memory, which is defined as the capacity of plants experiencing recurrent stress to “remember” past stressful events and prepare to respond in a better way when stressful conditions occur again (Bruce et al., 2007). Many terrestrial plants exposed to cyclic or episodic perturbations have shown increased tolerance when stress recur, a response referred to as hardening, priming, conditioning or acclimation (Zwiazek, 1991; Goh et al., 2003; Biber et al., 2009). This phenomenon includes by stress-induced structural, genetic and biochemical modifications that may lead to phenotypes with increased resistance (Baldwin and Schmelz, 1996; Bruce et al., 2007; Jaskiewicz et al., 2011; Yakovlev et al., 2011). It has been suggested that stress memory can last from several days to months and even years, and in some cases, it can be transmitted to the next generation (Baldwin and Schmelz, 1996; Iqbal and Ashraf, 2007; Rendina González et al., 2018).

Understanding of plant priming, including the length of plant stress memory as well as the mechanisms involved, remains largely unknown (Crisp et al., 2016). Molecular modifications are recognized as major mechanisms underlying stress memory in plants via activating, enhancing or speeding up responses to coping with environmental stressors (Crisp et al., 2016). Studies dealing with multiple stressors have also discovered an increasing number of epigenetic mechanisms responsible for the formation of stress memory in plants (Kinoshita and Seki, 2014; Dodd and Douhovnikoff, 2016; He and Li, 2018). Epigenetic modifications can alter gene expression without changing the underlying DNA sequence and occur in the form of DNA methylation, histone modifications and non-coding micro RNAs (Bossdorf et al., 2008; Bonasio et al., 2010). Epigenetic variations have the potential to increase phenotypic plasticity and accelerate adaptation to recurring stressful conditions (Verhoeven et al., 2016; Richards et al., 2017). DNA methylation is the most frequently studied and best understood epigenetic mechanism in plants. Several studies have revealed that environmental stress can result in an increase or decrease in cytosine-methylation throughout the genome and at specific loci to mediate environmentally-responsive and stress-responsive gene expression (Wada et al., 2004; Yaish et al., 2011; Dowen et al., 2012; Greco et al., 2013; Secco et al., 2015).

Seagrasses are a unique group of marine plants that have colonized the marine environment for thousands of kilometers of the sedimentary shorelines from sub-Artic to tropical regions over the past 60–90 million years ago (Les et al., 1997; Short et al., 2007). As foundation species of coastal ecosystems, seagrasses fulfill important ecosystem services including sediment stabilization, coastal protection, nutrient cycling, water quality improvement, fishery maintenance, and carbon sequestration, among others (Orth et al., 2006; Fourqurean et al., 2012; Nordlund et al., 2016). Despite their crucial functional role in the Earth ecosystem, seagrass meadows are declining due to rapid environmental changes driven by human activities (Orth et al., 2006; Waycott et al., 2009). Data from numerous studies across the globe have shown that seagrasses were disappearing worldwide at a rapid rate of 110 km^2^ per annum between the period of 1980 and 2006, which resulted in a loss of 29% of the total world seagrass population (Waycott et al., 2009). Indeed, ten seagrass species (∼14%) have already been listed at risk of extinction, while three species have been listed as endangered (Short et al., 2011). Some seagrass species are even predicted to go extinct by the end of this century, as is the case of the Mediterranean endemic *Posidonia oceanica*, as a consequence of warming trends and extreme oceanic events (Marbà and Duarte, 2010; Chefaoui et al., 2018). The situation is expected to worsen as a consequence of ongoing climate change (Waycott et al., 2009; Arias-Ortiz et al., 2018). One of the consequences of climate change in the marine environment is the ocean warming, a gradual increase in the mean of seawater temperature. However, climate change also gives way to extreme oceanic events (i.e., marine heatwaves), which have become conspicuous in the last few decades (Oliver et al., 2017, 2018). Marine heatwaves (MHW) are generally defined as extreme warm periods that last for at least 5 days with a level of temperature exceeding the 90th percentile, based on a three-decade historical baseline database (Hobday et al., 2016). In general, organisms have a lower capacity to overcome abrupt stress events rather than progressive ones. Thus, these extreme MHW may cause deleterious impacts on marine organisms that can result in shifts in species distributions and even local extinction (Easterling et al., 2000). The situation is predicted to worsen in the future with increasing evidence of more frequent, intense and longer-lasting MHW (Meehl and Tebaldi, 2004; Oliver et al., 2018; Darmaraki et al., 2019a). Indeed, a massive die-off of seagrass meadows has been reported after recent MHW, and in some cases had vast environmental consequences as the enormous amount of carbon dioxide stored in thousands of hectares of seagrass meadows were then released back to the atmosphere (Seddon et al., 2000; Arias-Ortiz et al., 2018).

As shown in terrestrial plants, epigenetic modifications and stress memory have the potential to provide responsive and adaptive mechanisms in seagrasses in order for them to withstand environmental changes (Davey et al., 2016; Duarte et al., 2018). As clonal plants, seagrasses provide a great opportunity to study the effects of epigenetics without concern about genetic variation. Nevertheless, our knowledge regarding the role of epigenetic modifications and stress memory remains unknown in seagrasses with only some experimental hints from transcriptomic studies (Marín-Guirao et al., 2017; Duarte et al., 2018; Marín-Guirao et al., 2019).

In an era of rapid global ocean changes, it is critical to better understand mechanisms driving seagrass thermal stress response in order to make timely decisions regarding seagrass conservation and management activities. Increasing our knowledge about the role of epigenetic modifications and stress memory can improve our recent predictions about the future of seagrasses (Marbà and Duarte, 2010; Jordà et al., 2012), enhancing our efforts to protect seagrasses worldwide. In this study, we simulated a scenario that will become more extreme and frequent in the future by conducting a two-heatwave experimental design for two Southern hemisphere seagrass species with different functional traits, *Posidonia australis* and *Zostera muelleri*. We hypothesized that plants pre-exposed to a stressful thermal event perform better and are less affected by subsequent heat stress than non-pre-heated plants. Plant responses were examined at different hierarchical levels including morphology, photo-physiology and gene expression in order to assess heat-stress induced priming effects on the two seagrass species. Regarding gene expression, special attention was paid to the response of methylation-related genes to explore the potential involvement of epigenetic modifications on seagrass heat-stress memory. A comparison between pioneer species with high morphological plasticity (*Z. muelleri*) and climax species with more stable and long-lived characteristics (*P. australis*) could help us to forecast the persistence of more or less stable communities under the future climate change scenarios.

## Materials and Methods

### Sample Collection

Fragments of *P. australis* and *Z. muelleri*, bearing several connected shoots were collected haphazardly at Port Stephens (PS) New South Wales (NSW), Australia (32°43′07.4″S 152°10′35.9′E) on the 19th of March 2019 and at Church Point (CP) NSW, Australia (33°38′46.8″S 151°17′11.9″E) on the 23rd of March 2019, respectively. Plant fragments were collected at a reciprocal distance >25 m in order to reduce the likelihood of sampling the same genotype twice. Both species were collected during low tide in shallow water (∼70 cm), then plant fragments were transported immediately to the seagrass mesocosm facility at the University of Technology Sydney (UTS). Environmental conditions including salinity and water temperature at PS and CP were measured at the same time as plant collection to mimic the natural conditions at the mesocosm facility at UTS. Water temperature was ∼25°C at both sites while the salinity was slightly higher at PS (34.1 ppt) than at CP (33.0 ppt). Rapid light curves were performed with a diving-PAM fluorometer (Walz GmbH, Germany) on three random plants at each site to define experimental light levels. These analyses showed that the saturating irradiance levels of plants in the field were approximately 350 μmol photons m^–2^ s^–1^ for both *P. australis* and *Z. muelleri* plants.

### Experimental Design

Once at UTS, plant fragments of both species with a similar number of shoots (i.e., 8–10 shoots) were carefully selected, individually planted into plastic trays filled with mini pebbles and randomly allocated in tanks of the mesocosm facility (three fragments per tank). In total, six aquaria were used for each species, 60-L aquaria for *P. australis* and 40-L aquaria for *Z. muelleri*. For each species, three experimental treatments including control (CT), treatment 1 heatwave (1HW) and treatment 2 heatwave (2HW) were conducted in parallel. Thus, for each treatment, two aquaria were considered as experimental replicates while six trays (fragments) were treated as biological replicates. Each aquarium was equipped with an independent light source (Hydra FiftyTwo HD^TM^, C2 Development, United States), two 55W-heaters and air and water pumps to maintain circulation and homogeneity of seawater temperature. For both species, the irradiance level was set at 350 μmol photons m^–2^ s^–1^ at canopy height according to the saturating levels of plants from the fields (mentioned above) with a 12 h:12 h light:dark period. Light cycle started from 7:30 a.m., with light levels progressively increasing to the maximum irradiance at 12:30 p.m. and kept for 2 h, before a progressive reduction until dark at 7:30 p.m. Water temperature was measured automatically every 30 min using iButton data logger (iButtonLink, United States) and manually checked twice a day using a digital thermometer (FLUKE 52II, United States). Throughout the experiment, purified water was added periodically to maintain the salinity level of 34 ppt and approximately 1/3 of seawater from each aquarium was renewed weekly to keep water quality consistent.

**FIGURE 1:**
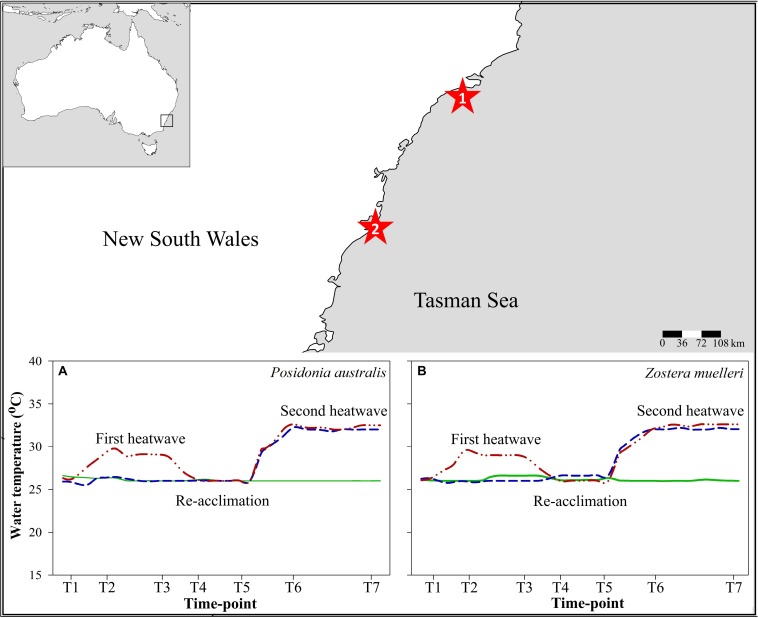
Sample collection sites during low tides: (1) Collection site of *Posidonia australis* at Port Stephens, New South Wales, Australia, (2) Collection site of *Zostera muelleri* at Church Point, New South Wales, Australia. Thermal profile in experimental treatments during the course of the experiment **(A,B)**: Green continuous lines: control; Blue dashed lines: Treatment 1-heatwave (1HW) and Red dashed lines with dots: Treatment 2-heatwave (2HW).

Water temperature was kept at 26°C (∼1°C above the temperature in natural conditions at the time of the experiment^1^) in all aquaria during a 2-week acclimation period (Figures 1A,B). Temperature was subsequently increased to 29°C (heating rate 1°C day^–1^) in two aquaria of 2HW of each species and maintained for 6 days to simulate a MHW. Water temperature in these heated tanks was then reduced to control levels to allow heated plants to re-acclimate during a 1-week period before simulating a second, more intense and longer-lasting MHW (32°C for 9 days; heating rate 1°C day^–1^). This second MHW was applied to four aquaria of each species, two pre-heated aquaria (2HW) and two non-pre-heated aquaria (1HW).

### Chlorophyll *a* Fluorescence

**FIGURE 2:**
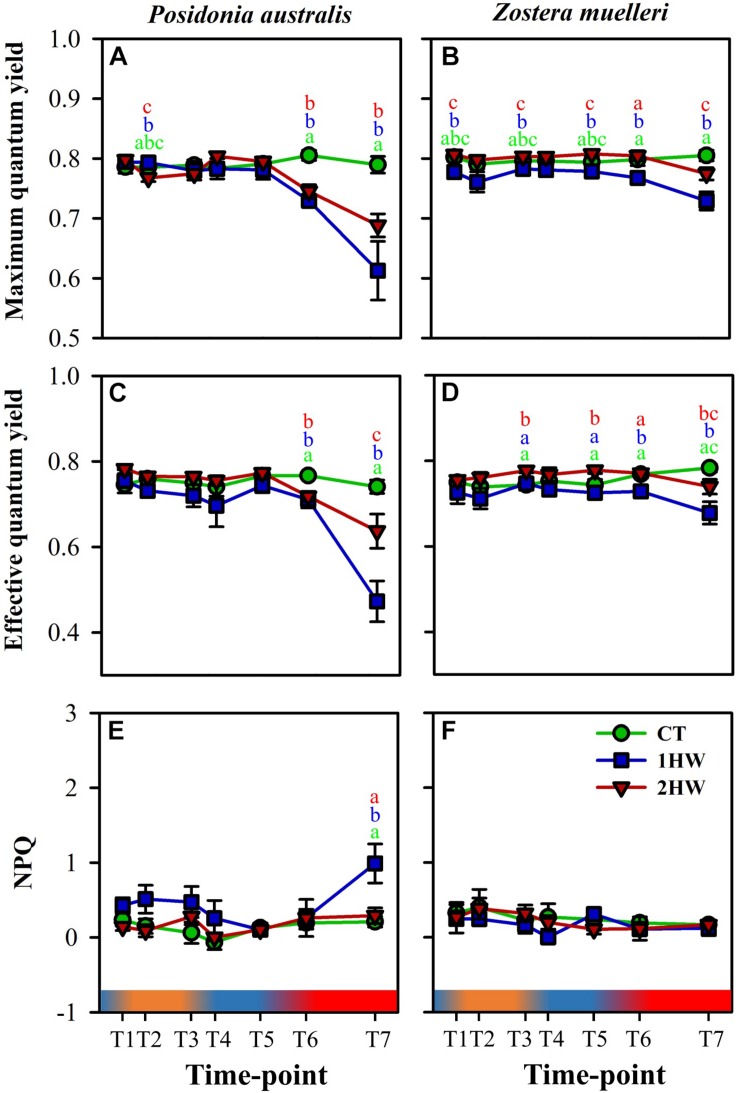
Photo-physiological reponses from Posidonia australis and Zostera muelleri: **(A,B)** Maximum quantum yield (*Fv/Fm*) were measured on dark-adapted plants; **(C,D)** Effective quantum yield (Δ*F/Fm*′) were measured on light-adapted plants and **(E,F)** Non-Photochemical Quenching (*NPQ*). CT: control, 1HW: 1 heatwave treatment, 2HW: 2 heatwaves treatment. At each time point, different colors correspond to different treatments (green – CT, blue – 1HW, and red – 2HW) and different letters (a-c) indicate significant differences between treatments [e.g. in Figure 2A: “a-green + b-blue + b-red” means CT ≠ 1HW = 2HW; Pair-wise comparison test, *p*_(perm)_ < 0.05]. Data are mean, ±SE, *n* = 5. Gradient bars present water temperature changes in treatment 2HW throughout the experiment.

The photophysiological response of *P. australis* and *Z. muelleri* plants was determined using a diving-PAM fluorometer following the methodology described elsewhere (Marín-Guirao et al., 2013). During the experiment, measurements were conducted on the second youngest leaf of five randomly selected plants from each treatment and each species at different time points along the course of the experiment (Figure 2): end of the first acclimation period – experiment started (T1); beginning of the first heatwave (T2); end of the first heatwave (T3); beginning of the re-acclimation period (T4); end of the re-acclimation period (T5); beginning of the second heatwave (T6) and end of the second heatwave – experiment ended (T7). Maximum quantum yield (*Fv*/*Fm*) of photosystem II (PSII) was measured on night dark-adapted plants (i.e., at 7 am, before start of light cycle) while the effective quantum yield of PSII (Δ*F*/*Fm*′) measurement was determined on light-adapted plants at noon during the daily period of highest irradiance level. Non-photochemical quenching (*NPQ*) was calculated according to the method of Maxwell and Johnson (2000) to estimate the amount of photosynthetic energy lost as heat (i.e., photo-protection).

### Plant Growth

Plant growth measurements were done by adopting the leaf marking method (Zieman, 1974). In the middle of the second acclimation period between both simulated heatwaves, five randomly selected plants of each treatment were marked just above the ligule. These samples were then collected at the end of the second heatwave (T7) for measuring leaf elongation (mm). Subsequently, newly grown leaf segments were dried at 70°C for 24 h and weighed to determine the growth as leaf biomass production (Dry weight).

### Pigment Contents

Approximately 50 mm from the middle portion of the second youngest leaf of *P. australis* and the whole second youngest leaf of *Z. muelleri* was harvested from five randomly selected plants of each treatment at the end of the experiment (T7) for analyzing pigments content. Collected leaf samples were cleaned of epiphytes and kept on ice before fresh weights were measured. Samples were homogenized in liquid nitrogen using pestles and mortars, transferred into 1.5 mL tubes containing 1 mL of 100% methanol and stored in complete darkness at 4°C for 8 h before centrifugation. Absorbance of 200 μL of obtained solution was read at 470, 652, 665, and 750 nm using a microplate reader (TECAN Infinite^®^ M1000 PRO, Switzerland) for calculations of the chlorophyll *a*, chlorophyll *b* and total carotenoid concentrations using equations from Wellburn (1994) after converting microplate readings into 1cm cuvette readings following Warren (2008) as described in Tran et al. (2018). Finally, results were normalized to a milligram of fresh weight.

### Quantitative Real-Time PCR (RT-qPCR)

#### Primer Design

Ten genes of interest (GOIs; Table 1) common to both species were chosen within three different categories including stress-related, photosynthesis-related and methylation-related genes.

**TABLE 1:** List of housekeeping genes and gene of interests used in this study.

Gene category	Gene name	Abbrev	Species	Forward primer (5′→3′)	Reverse primer (5′→3′)	Product size (bp)	E (%)	*R*^2^	Accession number	Reference (note)
Stress-related	Heat Shock Protein 90	HSP90	Zm	GAGGGTTTGTGCAAGGTCAT	GTTGGCAGTCCACCCATACT	123	103.9	0.996	ZM251873	This study
			Pa	TCAAGGAGGTGTCACACGAG	CAGATGCTCCTCCCAGTCAT	134	109.8	0.997	PO008787	This study
	Catalase	CAT	Zm	AAGTACCGTCCGTCAAGTGG	CTGGGATACGCTCCCTATCA	169	100.5	0.999	ZM230093	This study
			Pa							Same as *Z. muelleri*
	Manganese superoxide dismutase	MSD	Zm	TTTTCGCCAAGAACAAAACC	TCTGCATGATCTCTCCGTTG	135	99.8	0.998	ZM212939	This study
			Pa	AATAATGCCGCTCAGCTTTG	ACCCAACCAGATCCAAACAG	176	98.0	0.994	PO035322	This study
Photosynthesis-related	Photosystem II protein D1	psbA	Zm	AAGCTTATGGGGTCGCTTCT	GTGCAGCAATGAAAGCGATA	134	100.4	0.999	ZM045788	This study
			Pa	GACTGCAATTTTAGAGAGACGC	CAGAAGTTGCAGTCAATAAGGTAG	136	100.9	0.999	KC954695	Dattolo et al. (2014)
	Photosystem II protein D2	psbD	Zm							Same as *P. australis*
			Pa	CCGCTTTTGGTCACAAATCT	CGGATTTCCTGCGAAACGAA	161	103.6	0.999	KC954696	Dattolo et al. (2014)
	Rubisco large subunit	RBCL	Zm	CCGAGACAACGGCTTACTTC	AGTCATCTCGCGTTCACCTT	175	100.1	1.000	ZM194765	This study
			Pa	GCTGCCGAATCTTCTACTGG	CACGTTGGTAACGGAACCTT	177	102.2	0.999	U80719.1	Marín-Guirao et al. (2016)
Housekeeping	Glyceraldehyde 3-phosphate dehydrogenase	GAPDH	Zm	CGGTTACTGTAGCCCCACTC	CAAAGGCTGGGATTGGTTTA	79	100.8	0.992	Zoma_C_c6252	Kim et al. (2018)
			Pa	AGGTTCTTCCTGCTTTGAATG	CTTCCTTGATTGCTGCCTTG	138	110.3	0.998	GO347079	Serra et al. (2012)
	Elongation factor 1-alpha	Ef1A	Zm	AAGCAAAGGCGTCACTTGAT	TCTGCTGCCTTCTTCTCCTC	82	103.4	0.989	Zoma_C_c59090	Kim et al. (2018)
			Pa	GAGAAGGAAGCTGCTGAAATG	GAACAGCACAATCAGCCTGAG	214	107.2	0.997	GO346663	Serra et al. (2012)
	β-tubulin	TubB	Zm	GGACAAATCTTCCGTCCAGA	TCCAGATCCAGTTCCACCTC	185	102.8	0.995	Zoma_Contig120	Kim et al. (2018)
	Actin	Actin	Zm	TAAGGTCGTTGCTCCTCCTG	ACTCTGCCTTTGCAATCCAC	104	95.3	0.993	Zoma_ZMF02257	Kim et al. (2018)
	18S ribosomal RNA	18S	Pa	AACGAGACCTCAGCCTGCTA	AAGATTACCCAAGCCTGTCG	200	93.0	1.000	AY491942.1	Serra et al. (2012)
	Ubiquitin	UBI	Pa	CACCCTCGCTGACTACAACA	TTTCTCAGCCTGACGACCTT	195	97.2	0.998	GO347694	Serra et al. (2012)
Methylation- related	ProteinSet1/Ash2 histone methyl transferase complex subunit ash-2	ASH2L	Zm	CTCAGACCCCCAATTCTCAA	GTGGAAGAGACGACGGTGAT	153	100.3	0.994	ZM248014	This study
			Pa	CTATCCTGCTGCCTCCATGT	TCAACTGCACCTTCAACTCG	174	108.1	0.992	SRP126951	This study
	Histone-lysine N-methyltransferase setd3	SETD3	Zm	CGAACCTTCCTTTCTTGCTG	CCTCGGGTTGAGAATCAAAA	146	90.5	0.995	ZM228252	This study
			Pa	TGGGCTTGTGAACTGTGGTA	CGAATGATTGAGTCGTCCAG	200	103.9	0.949	SRP126951	This study
	Histone-lysine N-methyltransferase ATX2	ATX2	Zm	ATCCCGTGAATGTGGAGAAG	ATACCAGGCACCGTCGATAG	161	97.2	0.992	ZM254823	This study
			Pa	CCAGATACAAAGCTGCACCA	GCATTGTCATCCCCTTGAGT	170	103.1	0.993	SRP126951	This study
	Histone-lysine N-methyltransferase ATXR7 isoform X1	ATXR7	Zm	CAGAGGATCAATCCCTCCAA	CTTTGCCCGAACTCTTTCAG	138	102.0	0.990	ZM256759	This study
			Pa	CGAGTAGGGTCGAATGTGGT	ATCCATCCAGTCACACACGA	149	105.2	0.973	SRP126951	This study

*Pa: Posidonia australis, Zm: Zostera muelleri; E: Efficiency (%); R^2^: Calibration coefficient.*

*Zostera muelleri* GOIs were newly designed using *Z. muelleri* database from AquaticPlantsDB^®^ (Sablok et al., 2018),^2^ while housekeeping genes (HKGs) were taken from previous studies (Schliep et al., 2015; Pernice et al., 2016; Kim et al., 2018). For *P. australis*, however, no molecular resources are available to date, thus selected GOIs and HKGs were either newly designed or taken from previous studies on the congeneric species *P. oceanica*. Three photosynthesis-related genes (i.e., Photosystem II protein D1-psbA, Photosystem II protein D2-psbD and Rubisco large subunit-RBCL) and 4 HKGs were available in the literature (Serra et al., 2012; Dattolo et al., 2014; Marín-Guirao et al., 2016). The rest of the primers were designed using a *P. oceanica* transcriptome database available at the National Center for Biotechnology Information (NCBI) (Marín-Guirao et al., 2019).

Primers were designed using Primer3 v.0.4.0 (Koressaar and Remm, 2007; Untergasser et al., 2012) with the following default settings: primer lengths: 18–22 bp, product sizes: 100–200 bp and Tm = 59–61°C. Primers were validated for their specificities firstly by checking PCR amplification on agarose gel electrophoresis (i.e., only single band, similar size as designed) and secondly by checking the melting curve for each RT-qPCR run. RT-qPCR efficiencies were assessed via a series of cDNA dilutions of 384, 81, 27, 9, 3, and 1 ng using a linear regression model (Pfaffl, 2001). The efficiency of each primer pair was then calculated with the following equation: *E* (%) = (10^–1/slope^−1) × 100 (Radonić et al., 2004). Primers with efficiencies (*E*) within the range 90–110% and correlation coefficient >0.95 were used in the study (Table 1).

#### RNA Extraction and cDNA Preparation

Three leaf samples, targeted as a similar way for pigment content samples, were collected for RNA extraction at the end of each heatwave (T3 and T7). Epiphytes were carefully removed from plants and cleaned plant material was then immediately frozen in liquid nitrogen before being stored at −80°C until RNA extraction. PureLink^TM^ RNA Mini Kit (ThermoFisher, United States) was used to extract total RNA from both species. For *Z. muelleri*, extraction was done by following the manufacturer’s instructions. For *P. australis*, to minimize effects of phenolic compounds that can inhibit the extraction process, 2% (w/v) polyvinylpyrrolidone-40 (PVP) together with two glass beads were added to the lysis solution and vortexed at high speed at 4°C for 10 min, all other steps were completed by following the manufacturer’s instructions. During the extraction of total RNA, PureLink^TM^ DNase Set (ThermoFisher, United States) was added to eliminate genomic DNA. The total RNA quantity and quality were assessed with a NanoDrop spectrophotometer (ND-1000; NanoDrop Technologies, United States). Then, cDNA was synthesized from 500 ng of total RNA using the High-Capacity cDNA Reverse Transcription Kit (Applied Biosystems, United States) according to the manufacturer’s instructions. The resulting cDNA was diluted 1:20 prior to Reverse Transcription – quantitative Polymerase Chain Reaction (RT-qPCR) assays.

### Gene Expression Analyses

A 5 μL-final volume RT-qPCR reaction including 2.7 μL of iTaq^TM^ Universal SYBR^®^ Green Supermix (BIO-RAD, United States), 0.3 μL of 10 pmol μL^–1^ primers and 2 μL of diluted cDNA was robotically prepared in a 384-well PCR plate (BIO-RAD, United States) via an Automated Liquid Handling Systems (EpMotion^®^ 5075, Eppendorf, Germany). RT-qPCR assay was run in a Real-Time PCR Detection System (CFX384 Touch^TM^, Bio-Rad) with the following conditions: 95°C for 10 min, followed by 45 cycles of 95°C for 30 s, 60°C for 30 s and 68°C for 30 s. A melting curve from 60 to 95°C was also included for each amplicon to check the specificity of each reaction.

All RT-qPCR reactions were performed in three technical replicates with three no-template negative controls. Additionally, three No Reverse Transcription (No-RT) controls were prepared for each primer’s pair and included in each plate to ensure the absence effect of genomic DNA contamination (i.e., Cq value from No-RT sample was at least five cycles greater than the actual sample). Furthermore, an internal control assay was introduced in each plate to establish a reliable comparative result between different plates.

Data from RT-qPCR reactions were analyzed with Bio-Rad CFX Manager v3.1 software (BIO-RAD, United States) and normalized relative quantities of amplification were used to determine the changes in the gene expression level of GOIs as described in a previous study (Kim et al., 2018).

Before gene expression data analyses, three different algorithms were used to identify the best HKGs: NormFinder (Andersen et al., 2004), GeNorm (Vandesompele et al., 2002), and BestKeeper (Pfaffl et al., 2004).

Relative quantities of genes of interest (GOIs) were first normalized using the two best housekeeping genes selected from three different algorithms (Supplementary Data). Then, normalized data were used to determine gene expression levels of GOIs.

### Statistical Analyses

One-way analysis of variance (ANOVA; statistical software SPSS v.20) was used to check for significant differences in plant growth and pigment content between treatments at the end of the second heatwave (T7). Since these parameters greatly differ between the two species, each species was analyzed independently. Prior to the analysis, Levene’s test was used to check the homogeneity of variances and Shapiro–Wilk test was used to validate data normality. In case the parametric assumptions were not met, data were analyzed using Kruskal–Wallis test together with the Bonferroni correction for multiple tests (i.e., *P. australis*, Chl *b/a*, Table 3). A Tukey HSD *post hoc* test was applied whenever significant differences were determined.

Photo-physiological and gene expression results of GOIs were analyzed using Permutational Multivariate Analyses of Variance (PERMANOVA) on Primer 6 v.6.1.16 and PERMANOVA + v.1.0.6 software package (PRIMER-E Ltd) (Anderson et al., 2008). Analyses were performed on the resemblance matrices (created using Bray Curtis similarity) with a selected number of permutations of 9999. Within the analyses, treatment was treated as a fixed factor while time was treated as a random factor. Following, pair-wise test was performed to detect significant differences between treatments at each time point.

Principal component analyses (PCA) were also performed on normalized relative quantities of amplification of GOIs using the software PAST3 (Hammer et al., 2001) to determine responsive patterns to heat stress between treatment at each time point for gene expression data. Additionally, data from all measurements at T7 were analyzed all together using PCA to assess the difference in responses between the two seagrass species.

## Results

### Photo-Physiological Responses

During the first heatwave (T2-T3), neither of the species showed significant differences in *Fv*/*Fm* between heated (2HW) and non-heated (CT and 1HW) plants (Figures 2A,B), evidencing the absence of accumulated heat-damage at the PSII level. In fact, the photochemical efficiency of PSII (Δ*F/Fm*′) of heated plants was only slightly higher than that of control plants during this first heatwave (Figure 2), being significant only in *Z. muelleri* (CT = 1HW ≠ 2HW). The level of photo-protection (*NPQ*) of heated plants also showed no signs of alteration during this first warming exposure as seen by the lack of significant differences in *NPQ* between heated and control plants of both species (CT = 1HW = 2HW).

Contrarily, during the more intense and longer-lasting second heatwave (T6-T7), heated plants (1HW and 2HW) of both species experienced a significant reduction in their maximum and effective photochemical capacity of PSII (*Fv/Fm* and Δ*F/Fm*′) with respect to controls (Figures 2A–D), that resulted in significant differences between treatments over time [*p*_(perm)_ < 0.001, Table 2]. However, this heat-induced photochemical reduction was generally higher in non-preheated (1HW) than in preheated (2HW) plants of both species, and we found significant differences between non-preheated plants versus controls and preheated plants at T6 for both *Fv/Fm* and Δ*F/Fm*′ (Figures 2B,D; CT = 2HW ≠ 1HW). The differences between 1HW and 2HW plants were clear at T7. In *P. australis*, the second heatwave induced a 22% reduction in *Fv/Fm* and a 34% reduction in Δ*F/Fm*′ of 1HW plants while the reductions were much smaller in 2HW plants (13 and 14%, respectively). Differences were significant in Δ*F/Fm*′ (see Figure 2C, CT ≠ 1HW ≠ 2HW). Similarly, there was a significant reduction of 9% in *Fv/Fm* of *Z. muelleri*-1HW plants at T7, whereas there was only a slight reduction in *Fv/Fm* of 4% in *Z. muelleri*-2HW plants (Figure 2B, CT≠1HW≠2HW). We also observed a similar trend with Δ*F/Fm*′ results from *Z. muelleri*. In respect to CT plants, the reduction in Δ*F/Fm*′ in 1HW plants was more than double compared to that of 2HW plants (i.e., 14 and 6% respectively). Consequently, we found significant differences between plants from the two heating treatments (1HW and 2HW) as in case of *Fv/Fm* for *Z. muelleri* (Figure 2B; CT ≠ 1HW ≠ 2HW) and of Δ*F/Fm*′ for *P. australis* (Figure 2C; CT ≠ 1HW ≠ 2HW).

**TABLE 2:** PERMANOVA analysis performed on photo-physiological measurements assessing the effect of increased seawater temperature among different treatments overtime.

Species	Measurement	Source	df	SS	MS	Pseudo-F	*p*_(perm)_	Unique perms
*Posidonia australis*	Maximum quantum yield	Time	6	537.87	89.646	12.632	**0.0001**	9946
		Treatment(Time)	14	516.3	36.879	5.1968	**0.0001**	9925
	Effective quantum yield	Time	6	1468	244.66	15.354	**0.0001**	9947
		Treatment(Time)	14	1497.5	106.97	6.7128	**0.0001**	9928
	NPQ	Time	6	713.35	118.89	3.3991	**0.0045**	9945
		Treatment(Time)	14	1084.3	77.448	2.2142	**0.0129**	9907
*Zostera muelleri*	Maximum quantum yield	Time	6	33.814	5.6357	3.5623	**0.0037**	9932
		Treatment(Time)	14	122.68	8.7628	5.5389	**0.0001**	9930
	Effective quantum yield	Time	6	36.221	6.0368	1.2295	0.2884	9938
		Treatment(Time)	14	264.19	18.871	3.8433	**0.0002**	9924
	NPQ	Time	6	203.36	33.893	1.0434	0.3953	9944
		Treatment(Time)	14	235.6	16.828	0.51807	0.9169	9918

*Significant differences [p(perm) < 0.05] are in bold.*

Regarding non-photochemical quenching (NPQ), *Z. muelleri* interestingly showed no significant differences [*p*_(perm)_ = 0.9169, Pseudo-*F* = 0.5181, Table 2] among three treatments throughout the whole experiment (Figure 2E). In contrast, *P. australis*-1HW plants significantly tripled their NPQ levels at T7 compared to CT and 2HW plants [Figure 2F, Treatment (Time): *p*_(perm)_ < 0.001, Pseudo-*F* = 0.5181, Table 2, CT = 2HW ≠ 1HW].

### Plant Growth Responses

**FIGURE 3:**
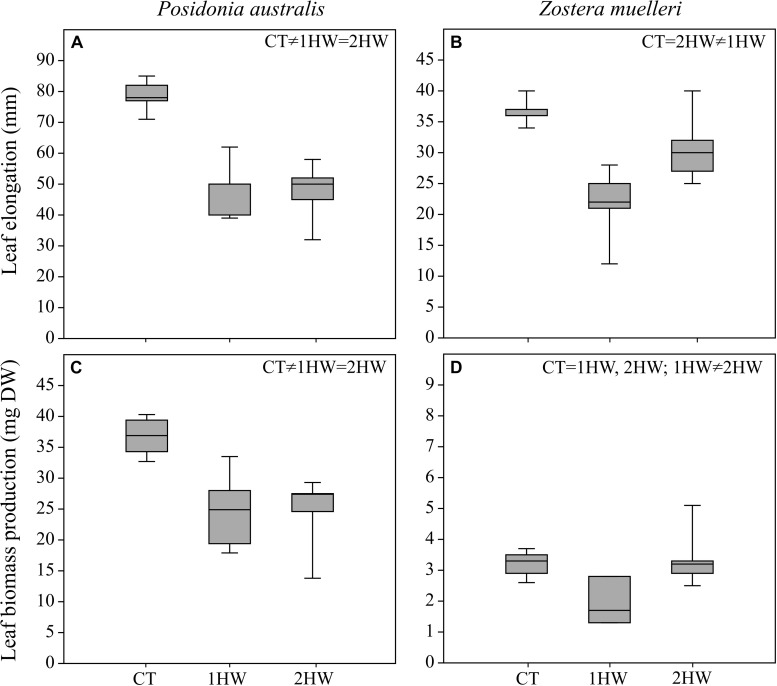
Leaf elongation **(A,B)** and leaf biomass production (Dry weight; **(C,D)** from control (CT), non-pre-heated (1HW) and pre-heated (2HW) plants at the end of the second heatwave (T7). Tukey HSD *post hoc* results are shown on the top of the graphs (Significant difference means *p* < 0.05). Data are mean, *n* = 5, ±SE.

Increased temperatures during the second heatwave (32°C) significantly reduced leaf elongation and leaf biomass production of both preheated (2HW) and non-preheated (1HW) *P. australis* plants (Figures 3A,C; *p* < 0.01, Table 3). Growth reduction, however, was similar in 2HW plants (39%) and 1HW plants (40%).

**TABLE 3:** Results from One-way ANOVA analyses and Kruskal–Wallis test performed on plant growth and pigment content results.

Species	Measurement	Statistical analysis	df	*F*	*p*
*Posidonia australis*	Biomass	One-way ANOVA	2	8.130	**0.006**
	Leaf growth	One-way ANOVA	2	22.459	**0.000**
	Chl *a*	One-way ANOVA	2	3.698	0.056
	Chl *b*	One-way ANOVA	2	2.161	0.158
	Chl *b/a*	Kruskal–Wallis test	2		**0.007**
	Carotenoids	One-way ANOVA	2	1.301	0.308
*Zostera muelleri*	Biomass	One-way ANOVA	2	4.959	**0.027**
	Leaf growth	One-way ANOVA	2	11.473	**0.002**
	Chl *a*	One-way ANOVA	2	0.893	0.435
	Chl *b*	One-way ANOVA	2	0.041	0.960
	Chl *b/a*	One-way ANOVA	2	16.767	**0.000**
	Carotenoids	One-way ANOVA	2	0.795	0.474

*Significant differences (p < 0.05) are in bold.*

In *Z. muelleri* plants, significant differences among treatments (*p* < 0.05, Table 3) were also detected for both leaf elongation and leaf biomass production measurements. During the second heatwave, leaf elongation rate decreased by 41% in 1HW plants while there was only a 16% reduction in the case of 2HW plants (Figure 3B; CT = 2HW ≠ 1HW). It is interesting to note that while leaf biomass production decreased by 38% in 1HW plants, 2HW plants accumulated 6% more biomass than the CT plants during the second heatwave (Figure 3D). This phenomenon led to a significant difference between 1HW vs. 2HW plants in terms of leaf growth (Figure 3D; CT = 1HW, 2HW; 1HW ≠ 2HW).

### Pigment Content Responses

**FIGURE 4:**
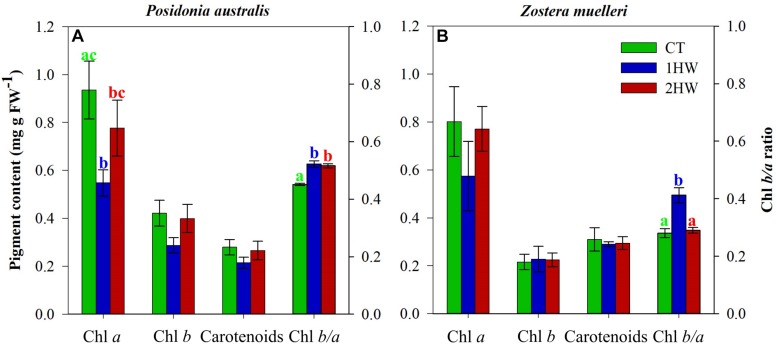
Pigment relations at the end of the second heatwave (T7): Chlorophyll *a* (Chl *a*), Chlorophyll *b* (Chl *b*), Carotenoids and the Chlorophyll *b*/*a* molar ratio (Chl *b*/*a*) in *P. australis* **(A)** and Z. muelleri **(B)**. CT = control plants; 1HW = non-pre-heated plants; 2HW = pre-heated plants. Different letters (a–c; green letters correspond with CT, blue letters correspond with 1HW and red letters correspond with 2HW treatment) indicate significant differences (*p* < 0.05) among treatments as derived from Tukey HSD *post hoc* analyses. Error bars present ±SE, *n* = 5.

Chlorophyll *a* appeared as the most sensitive photosynthetic pigment to temperature increase among pigments measured at the end of the experiment, both in *P. australis* and in *Z. muelleri* (Figures 4A,B). Interestingly, 2HW plants were able to maintain their Chl *a* contents similar as in CT plants, while 1HW plants suffered a strong reduction (41 and 28% for *P. australis* and *Z. muelleri*, respectively). Via Tukey HSD *post hoc* test, we found a significant difference between 1HW plants and CT plants in *P. australis* (Figure 4A). Both Chl *b* and Carotenoids content (Table 3) from 1HW *P. australis* plants were further impacted by elevated temperature during the second heatwave when compared to those from 2HW plants (Figure 4A), although these differences were not statistically significant.

Temperature increase affected Chl *a* and Chl *b* contents differently of the two seagrass species, contributing to significant differences in Chl *b/a* ratios among experimental treatments (*p* < 0.01, Table 3). In *P. australis*, both 1HW and 2HW plants increased ∼13% of Chl *b/a* ratios in respect to the CT plants (Figure 4A). In contrast, only non-preheated (1HW) *Z. muelleri* plants increased their Chl *b/a* ratios (32% more than in CT plants) significantly, while preheated plants kept their Chl *b/a* ratios comparable to control levels (0.28 and 0.29 in CT and 2HW plants, respectively; Figure 4B).

### Gene Expression Responses

All primers were tested in the two species and some of them successfully worked on both *P. australis* and *Z. muelleri* (i.e., psbD and CAT, Table 1), indicating the presence of conservative genomic regions between the two different seagrass species belonging to different genera.

**FIGURE 5:**
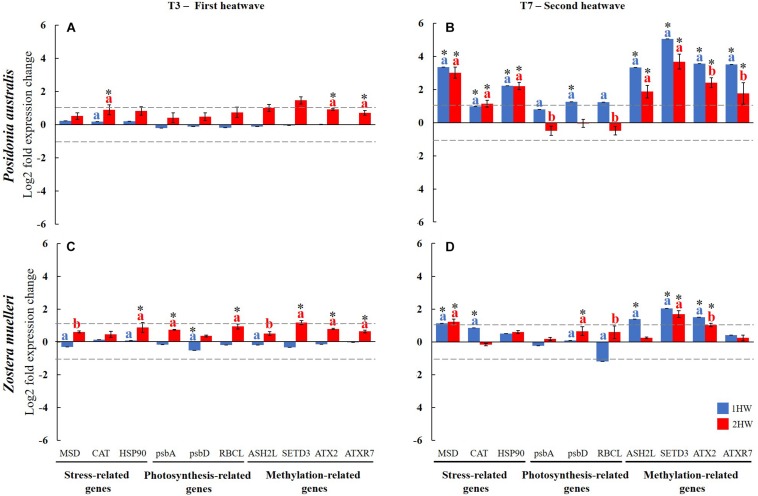
Differential gene expression for GOIs at the end of the first (T3; left panels) and second heatwaves (T7; right panels), respectively. For *P. australis* **(A,B)** and *Z. muelleri* **(C,D)**. Data is expressed as log2 Relative Quantification versus the control group. Data are mean, ±SE, *n* = 3. Pair-wise results are presented on top of the column corresponding to significant difference between control and treatments (asterisk) or between the two treatments (letters), *p* < 0.05. 1HW: 1 heatwave plants; 2HW: 2 heatwave plants.

In general, during the first heatwave (T3), 2HW plants from both species showed up-regulation of all analyzed GOIs with respect to plants under control temperature (CT and 1HW). The difference, however, was significant only for 3 and 6 genes in *P. australis* and *Z. muelleri*, respectively (Figures 5A,C).

At the end of the second heatwave (T7), all heated *P. australis* plants (1HW and 2HW) activated substantial molecular response to compensate with extreme temperature changes, with 80% of the GOIs tested showing significant up-regulation (Figure 5B). In *Z. muelleri*, while we observed a similar number of significantly affected genes at T3 and T7 (Figure 5C), the GOIs significantly regulated were different between the two time points. In both species, results from both T3 and T7 confirmed methylation-related genes were more sensitive to temperature increase than stress-related and photosynthesis-related genes. Details about statistical analysis results from each GOIs at T3 and T7 can be found in Table 4.

**TABLE 4:** PERMANOVA analysis performed on gene expression levels of GOIs from different treatments.

		*Posidonia australis*						*Zostera muelleri*					
GOI	Source	df	SS	MS	Pseudo-F	*p*_(perm)_	Unique perm	df	SS	MS	Pseudo-F	*p*_(perm)_	Unique perm
HSP90	Time	1	11370	11370	54.839	**0.0001**	9950	1	1367.2	1367.2	5.708	**0.023**	9946
	Treatment(Time)	4	9144.3	2286.1	11.026	**0.0001**	9928	4	2341.1	585.3	2.444	0.074	9952
CAT	Time	1	1654.8	1654.8	7.715	**0.0059**	9943	1	661.2	661.2	5.783	**0.032**	9932
	Treatment(Time)	4	3888.5	972.13	4.5323	**0.0052**	9944	4	2475.7	618.9	5.413	**0.009**	9945
MSD	Time	1	13097	13097	31.569	**0.0001**	9958	1	5659.8	5659.8	80.129	**0.000**	9956
	Treatment(Time)	4	11668	2917	7.0308	**0.0001**	9933	4	4383.4	1095.8	15.514	**0.000**	9937
psbA	Time	1	3337.6	3337.6	9.5367	**0.0009**	9939	1	1683.3	1683.3	9.690	**0.002**	9946
	Treatment(Time)	4	3082.5	770.63	2.2019	**0.0762**	9942	4	1797.0	449.2	2.586	**0.054**	9956
psbD	Time	1	6433.2	6433.2	23.064	**0.0001**	9930	1	446.1	446.1	2.997	0.099	9911
	Treatment(Time)	4	3889.9	972.47	3.4865	**0.0081**	9943	4	2147.8	536.9	3.607	**0.019**	9964
RBCL	Time	1	9830.1	9830.1	40.425	**0.0001**	9952	1	1219.8	1219.8	3.474	0.061	9948
	Treatment(Time)	4	5831.5	1457.9	5.9954	**0.0001**	9929	4	6060.9	1515.2	4.315	**0.004**	9938
ASH2L	Time	1	7703.7	7703.7	16.493	**0.0001**	9960	1	1789.8	1789.8	33.712	**0.000**	9945
	Treatment(Time)	4	12466	3116.6	6.6724	**0.0001**	9935	4	4312.0	1078.0	20.305	**0.000**	9954
SETD3	Time	1	8866.8	8866.8	13.863	**0.0001**	9953	1	217.0	217.0	1.685	0.196	9939
	Treatment(Time)	4	18997	4749.3	7.4254	**0.0001**	9923	4	10304.0	2576.0	19.997	**0.000**	9954
ATX2	Time	1	13600	13600	64.66	**0.0001**	9960	1	541.8	541.8	9.199	**0.002**	9953
	Treatment(Time)	4	14096	3523.9	16.754	**0.0001**	9942	4	5489.7	1372.4	23.301	**0.000**	9956
ATXR7	Time	1	5666.8	5666.8	14.48	**0.0001**	9951	1	128.4	128.4	1.680	0.226	9928
	Treatment(Time)	4	11148	2786.9	7.1212	**0.0001**	9942	4	1318.4	329.6	4.312	**0.015**	9950

*Significant differences [p(perm) < 0.05] are in bold.*

#### Methylation-Related GOIs

At T3, heated plants of both species (2HW) showed significant increased transcripts accumulation of ATX2 and ATXR7 (CT = 1HW ≠ 2HW). ASH2L was also highly up-regulated in heated plants although without significant differences among treatments (Figures 5A,C). We also found a significant upregulation of SETD3 in *Z. muelleri* heated plants during the first heatwave (Figure 5C).

At T7, most methylation-related GOIs showed significant up-regulations in 1HW and 2HW heated plants of both species (Figures 5B,D). Significant differences between 1HW and 2HW *P. australis* plants were found in ATX2 and ATXR7 (Figure 5B, 1HW > 2HW). *Z. muelleri* plants followed a similar trend, with 1HW plants showing higher gene expression levels than 2HW plants among all methylation-related GOIs with significant differences found for ASH2L and ATX2 (Figure 5D).

#### Stress-Related GOIs

At T3, positive changes were observed in all stress-related and photosynthesis-related GOIs, with significant up-regulations (CT = 1HW ≠ 2HW) detected in CAT from *P. australis* plants (Figure 5A) and in HSP90 from *Z. muelleri* plants (Figure 5C).

At T7, for *P. australis*, the three stress-related GOIs (i.e., MSD, CAT, and HSP90) showed similar and significant up-regulation in all heated plants (1HW and 2HW) (Figure 5B, CT ≠ 1HW = 2HW). In contrast, CAT showed a significant difference between the two categories of heated *Z. muelleri* plants (1HW > 2HW, Figure 5D).

#### Photosynthesis-Related GOIs

At T3, all photosynthesis-related GOIs showed up-regulations in heated (2HW) plants of both studied species, although significant differences (CT = 1HW ≠ 2HW) were only detected in *Z. muelleri* plants for psbA and RBCL (Figure 5C).

At T7, non-preheated *P. australis* plants (1HW) increased their levels of gene expressions significantly compared to CT plants (CT ≠ 1HW in psbD), while preheated-plants (2HW) maintained or even decreased the expression levels of those genes, resulting in significant differences between the two heated plants among all photosynthesis-related GOIs (1HW ≠ 2HW, Figure 5B). In contrast, in *Z. muelleri*, no significant difference was found between 1HW and 2HW plants in cases of psbA and psbD (1HW = 2HW, Figure 5B). Moreover, even if no significant difference was detected between CT versus heated plants (CT = 1HW = 2HW), RBCL was expressed differently between 1HW and 2HW plants. As a consequence, the expression levels of RBCL was significantly different between the two heated treatments at T7 (1HW ≠ 2HW).

**FIGURE 6:**
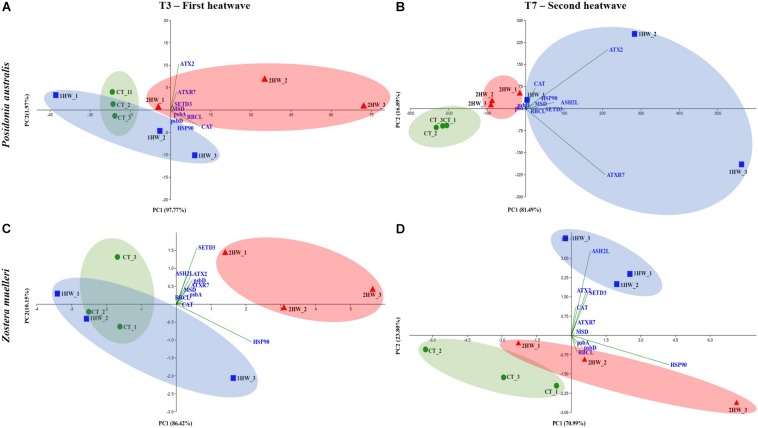
PCAs conducted on gene expression data. **(A)** *Posidonia australis* at T3, **(B)** *P. australis* at T7, **(C)** *Zostera muelleri* at T3 and **(D)** *Z. muelleri* at T7. Different colors correspond to different treatments (Green circle = Control-CT, Blue square = Treatment 1-heatwave-1HW, Red triangle = Treatment 2-heatwave-2HW).

Principal component analyses performed on gene expression results from both seagrass species demonstrated clearly that: (***a***) at T3, heated plants (2HW) were separated from non-heated plants (CT and 1HW) while (***b***) at T7, the two groups of plants experiencing heat stress (1HW and 2HW) were distant from CT plants, with 2HW plants showing more similarities to CT plants than to 1HW plants (Figure 6). PCA results also highlighted methylation-related genes were the main drivers differentiating 2HW plants at T3 and 1HW plants at T7. For instance, in *P. australis* at T3, ATX2 and ATXR7 together with CAT were the main drivers separating 2HW plants away from CT and 1HW plants along the PC1 *axis* responsible for 97.77% of this separation (Figure 6A). Whilst, in *Z. muelleri*, SETD3 and HSP90 mainly contributed to PC1, which was responsible for 86.42% of the separation between 2HW plants with the other two groups (Figure 6C). At T7, in *P. australis* ATX2 and ATXR7 remained the strongest factors separating 1HW plants from 2HW and CT plants (Figure 6B) while in *Z. muelleri*, ASH2L together with ATX2 and SETD3 separated 1HW plants from CT and 2HW plants along PC2 (23.8%) (Figure 6D).

**FIGURE 7:**
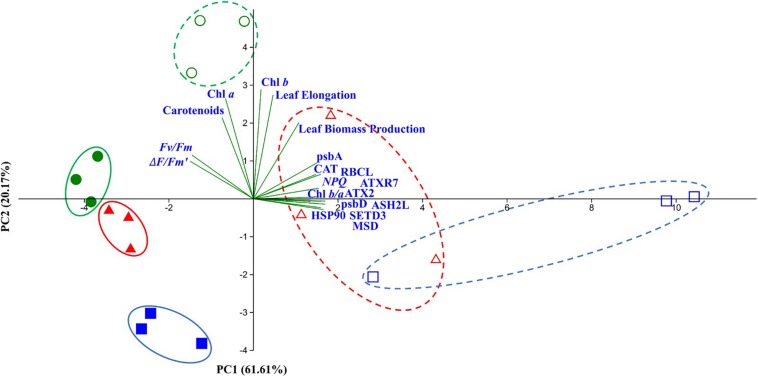
PCA conducted on morphological, physiochemical and gene expression data at T7. Different colors and shapes correspond to different treatments (Green circle = Control-CT, Blue square = Treatment 1-heatwave-1HW, Red triangle = Treatment 2-heatwave-2HW) and species (filled = *Zostera muelleri*, un-filled = *Posidonia australis*).

Principal component analyses results for both species and all analyzed plant variables at T7 showed similar results in both seagrass species with heated plants separated from control plants, reflecting the overall effects (i.e., molecular, physiological and organismal effects) of extreme temperature increase during the second heatwave (Figure 7). Nonetheless, preheated-plants (2HW) were closer to control plants than non-preheated ones, especially in the case of *Z. muelleri*. Additionally, control plants of both species were located within the same quadrat II of the PCA graph (Figure 7), in accordance with their higher photochemical capacity (*Fv/Fm*; Δ*F/Fm*′) and pigments content (Chl *a* and carotenoids). In contrast to controls, heated plants of the two species were separated along PC1 *axis* (responsible for 61.61% of total variance; Figure 7), suggesting slight differences in the response of the two seagrass species to the experimental recurrent heatwave at T7.

## Discussion

This comparative experiment involving *P. australis* and *Z. muelleri* provided us with a unique opportunity to better understand the thermal tolerance of two contrasting functional types of seagrass species from the southern hemisphere. Results from molecular to organismal levels support the fast-growing - pioneer *Z. muelleri* to be more tolerant than the long-lived - climax *P. australis*. In addition, by including a two-heatwave experimental design, we demonstrated that pre-heated plants performed better during the more extreme second heatwave, suggesting that they might have acquired mild stress-induced traits during the first heatwave. These results provided the very first insight into thermal hardening in seagrasses. Furthermore, gene expression analyses supported a key role of methylation-related genes in the responses of these two seagrass species to thermal stress, suggesting the importance of epigenetic modifications on seagrass memory and response to changing environment.

### Difference Between Climax Versus Pioneer Seagrass Species in Response to Thermal Stress

Photo-physiological results showed that both *P. australis* and *Z. muelleri* were more affected during the second heatwave (T6-T7) than during the first heatwave (T2-T3). This observation was expected since the second HW was more intense and longer-lasting than the first heatwave. On the other hand, the greater photochemical inhibition of heated-*P. australis* in comparison with heated-*Z. muelleri* (Figure 2), indicated interspecific differences in heat tolerance. Our photo-physiological results concur with previous studies on Mediterranean seagrass species (i.e., *Posidonia oceanica* and *Cymodocea nodosa*), showing the climax more stable species further suffer from negative effects of thermal stress rather than the fast-growing pioneer species (Marín-Guirao et al., 2016, 2018). It is important to note that 1HW-*P. australis* activated Non-Photochemical Quenching (NPQ) machinery, a photo-protective mechanism commonly used by plants to overcome stressful conditions (Ashraf and Harris, 2013). Contrarily, neither 2HW nor 1HW-*Z. muelleri* changed their NPQ values during the second heatwave. With the fact that 1HW-*Z. muelleri* suffered a significant reduction in both maximum quantum yield of PSII (*Fv/Fm*) and effective quantum yield capacities (Δ*F/Fm*′) at T7 (Figures 2B,D), these results suggest that *Z. muelleri* plants went through a different pathway or initiated a different mechanism to protect their photosynthetic organelles from photo-damaging when exposed to heat stress.

In contrast to *Z. muelleri*, evidences of Chlorophyll *a* (Chl *a*) degradation were obtained for non-pre-heated *P. australis* plants at the end of the second heatwave. This reduction in pigments content was congruent with the greater photochemical alterations detected in *P. australis* during the second heatwave with regard to *Z. muelleri*. During the stressful condition, the degradation of Chl *a* might suggest that (***a***) Chl *a* was damaged by the higher temperature and/or (***b***) it is a response to modify the light harvesting capacity since changes in Chl *a* give a rise to changes in the Chl *b*/*a* ratio which is a proxy of PSII antenna size. Our results support previous work by York et al. (2013), showing a minor effect of temperature increase on modifying photosynthetic pigments in *Z. muelleri*. Interestingly, for *P. australis*, our results differed with the ones previously obtained for a closely related species from the same genus (i.e., the Mediterranean endemic seagrass *P. oceanica*) (Marín-Guirao et al., 2016, 2018). Marín-Guirao et al. (2018) did not find evidence of warming-induced pigment alterations after heat exposures of different intensity and duration from *P. oceanica* plants from different thermal origins. In contrast, in our study, we found negative effects of temperature increase on pigments content in *P. australis* with great reductions (especially in 1HW plants) in all pigment parameters. These contrasting findings could be explained by evolutionary and local adaptations that could also have played an important role in differentiating these two sister species (King et al., 2018).

Gene expression analyses provided more clues about the interspecific differences between the two species at the molecular level. As seen in many previous studies in seagrasses (Bergmann et al., 2010; Winters et al., 2011; Marín-Guirao et al., 2016, 2017; Tutar et al., 2017; Mota et al., 2018; Traboni et al., 2018; Nguyen et al., 2020), heat stress commonly yielded a high expression level of stress-related genes (e.g., HSP90, CAT) and photosynthesis-related genes (e.g., psbA and psbD). Similarly, we also detected significant up-regulation among our GOIs from the same categories during the second heatwave (T7) from both tested species. However, the differences in *P. australis* between heated and control plants were, in most cases, the double of the differences found in *Z. muelleri*. This could indicate that the applied thermal treatment induced a greater stress level to *P. australis* that, in consequence, required a stronger molecular response to compensate for the heat-stress experienced during the second heatwave.

Principal Component Analyses performed on all collected data at T7 (Figure 7), showed the differences in the response to heat stress between *P. australis* and *Z. muelleri.* Importantly, while photosynthetic-related factors (e.g., *Fv/Fm*, Chl *a*) were the main drivers differentiating *Z. muelleri*, the rest of measured parameters (e.g., GOIs, biomass) were responsible for *P. australis*.

All the differences from a molecular level, pigment contents to photo-physiology were translated to higher growth reductions in *P. australis* than in *Z. muelleri* as seen in Figure 3. These results clearly reflect the higher heat sensitivity of the climax species and are in agreement with previous studies in the Mediterranean, which have also shown greater growth reduction from heat stress for the climax *P. oceanica* compared to the pioneer *Cymodocea nodosa* (Olsen et al., 2012; Marín-Guirao et al., 2018; Savva et al., 2018). Together with previous studies, our study strongly demonstrates that the climax seagrass species (e.g., *P. oceanica* and *P. australis*) will likely suffer from ocean warming in the coming decades, while some pioneer species (e.g., *C. nodosa* and *Z. muelleri*) may be more tolerant and might even benefit from future-warmer oceans.

### Thermal Priming Effects on Seagrasses

Our study provides, for the first time, some evidence for thermal priming effects in seagrasses. Looking at the photo-physiological results at the second heatwave, it is clear that 2HW plants had been primed during the first heatwave (Figure 2). From both tested seagrass species, *Fv/Fm* and Δ*F/Fm*′ values were higher (significantly in some cases) in preheated plants (2HW) than in non-preheated ones (1HW). Studies from terrestrial plants (Smillie and Gibbons, 1981; Wang et al., 2014; Li et al., 2015) have similarly shown that primed plants had a higher photosynthetic rate in relation to the non-primed plants. Hence, our photo-physiological results strongly support priming effects on studied seagrass species from a photosynthetic point of view. Focusing on T7, while the 2HW-*P. australis* were able to keep their NPQ values similar to CT plants – indicating priming for the heatwave, in contrast, the 1HW-*P. australis* greatly increased their NPQ as a common photo-protective mechanism in stressed plants (Ashraf and Harris, 2013).

From a morphological perspective, we also detected significant differences between un-primed- (1HW) and primed-(2HW) *Z. muelleri* in terms of leaf elongation and leaf biomass production (Figure 3). For both parameters, 1HW-*Z. muelleri* suffered a significant reduction with respect to 2HW plants and CT plants as well. This indicated that 2HW plants were primed by the first heatwave, performed better during the second heatwave and were able to better maintain their growth as compared to that of the 1HW plants. Our results are similar to those from terrestrial plants (Wang et al., 2014) that also showed that primed *Triticum aestivum L.* maintained their biomass compared to un-primed plants during a more severe high-temperature stress. It is likely that the relatively slow growth rates of this climax species and the short marking time (i.e., growing period) compared to the pioneer species, did not allow for the detection of differences in growth between both heat treatments (1HW vs 2HW). As a result, we believe a longer growing period would be needed to detect a growth change.

In support of our hypothesis of thermal priming effects in seagrasses, large Chl *a* reductions were only detected in leaves of non-pre-heated plants (1HW). This becomes more obvious in the Chl *b/a* ratios of *Z. muelleri* at the end of the second heatwave (T7). While pre-heated 2HW plants kept their Chl *b/a* ratios similar to the controls, non-pre-heated 1HW plants experienced a significant increase in Chl *b/a* ratios as seen in previous studies in terrestrial plants (Almeselmani and Viswanathan, 2012; Niu et al., 2017).

At the molecular level, there were more indications that priming had an effect on both species. This is indicated by a significantly lower expression level of some GOIs from 2HW plants compared to those from 1HW plants. In *P. australis* at T7, the expression levels of some methylation-related GOIs (i.e., ATX2 and ATXR7) and photosynthesis-related GOIs (i.e., psbD) were significantly higher in non-preheated plants (1HW) in comparison with preheated plants (2HW) and control plants (CT). Similarly, more evidence supporting the thermal priming hypothesis can also be found in stress-related GOIs (i.e., CAT) and methylation-related GOIs (i.e., ASH2L and ATX2) in heated *Z. muelleri*.

In addition, our PCA results at T7 (Figures 6B,D, 7) further support the priming effects by showing, in both studied species, that 2HW plants were clustered with CT plants while 1HW plants were more separated away from those two former groups.

During the first heatwave (T3) the two species showed differences in gene expression. While a large amount of GOIs (i.e., 6/10) showed significant up-regulation in *Z. muelleri*, only 3 GOIs were significantly up-regulated in *P. australis*. An alternative to epigenetic modifications, the accumulation of protective molecules (i.e., HSPs) is also likely involved in facilitating a fast stress response and hence are also possible mechanisms underlying stress memory. At T3, only *Z. muelleri* activated HSP90 which is a well-known heat-protective molecule also involved in the heat stress response of different seagrasses (Marín-Guirao et al., 2016; Tutar et al., 2017; Mota et al., 2018; Traboni et al., 2018). Together, these differences between the two species suggest that *Z. muelleri* plants were, indeed, more prone to thermal priming and hence to acquire thermal tolerance after recurrent heat events than *P. australis* plants.

Our study also suggested the involvement of methylation-related genes or epigenetic modifications in response to thermal stress in seagrasses. Our results, indeed, confirmed recent transcriptomic discoveries in seagrasses showing the induction of genes involved in DNA and histone methylation, including our methylation-related GOIs (i.e., ATX2 and SETD3), in heated *P. oceanica* (Marín-Guirao et al., 2017, 2019). Among our methylation-related GOIs, ProteinSet1/Ash2 histone methyl transferase complex subunit ash-2 (ASH2L) and Histone-lysine N-methyltransferase ATX2 are known as being specifically involved in methylation and dimethylation at Lys4 of histone H3 (H3K4) (Wysocka et al., 2003; Patel et al., 2009). Methylation status of H3K4 has been shown to be involved in changing chromatin structure during environmentally-induced transcriptional memory (D’Urso and Brickner, 2017) and plant stress response via activating or silencing gene expression (Shanker, 2016). In addition, ATXR7 belongs to the Trithorax family proteins that connect with seasonal memory in plants (Iwasaki and Paszkowski, 2014). On the other side, Histone-lysine N-methyltransferase SETD3 is linked to H3K36 methyltransferase (Pontvianne et al., 2010; Suzuki et al., 2017) which in plants has been suggested to play an important role in development and stress responses (Huang et al., 2016). The regulations of the methylation-related GOIs in our study are consistent with previous work which highlighted the role of epigenetic modifications in seagrasses (Davey et al., 2016; Marín-Guirao et al., 2017; Duarte et al., 2018; Marín-Guirao et al., 2019) or in terrestrial plants (Chinnusamy and Zhu, 2009; Liu et al., 2015; Rey et al., 2016).

### Future Perspectives

While our study demonstrates, for the first time, thermal priming effects on two seagrass species from the southern hemisphere, the duration of our experiment was relatively short in comparison to what the plants experience in their natural environment (i.e., marine heatwaves, see Hobday et al., 2016). For that reason, more ecologically relevant studies (e.g., Olsen et al., 2012; York et al., 2013) on stress memory in seagrasses are needed to confirm and broaden our findings. Moreover, considering that local adaptation could be responsible for many inter- and intra-specific differences among different species and different seagrass populations (Procaccini et al., 2007; King et al., 2018), together with the fact that we used only one population from each species, future studies clearly need to investigate more species and more populations in order to complete our knowledge on thermal priming effects on seagrasses.

Another point that should be considered in future studies is the importance of testing the length (duration) of the stress memory since the adaptive success of the species could be highly dependent on this factor. Recently, one study from the Baltic Sea has shown methylation patterns of *Zostera marina* changed under heat stress conditions and importantly, the seagrass did not return to pre-stress patterns after a 5.5-week recovery period (Jueterbock et al., 2019). This could explain why gene expression levels of methylation-related GOIs of 2HW plants were relatively lower than those from 1HW plants during the second heatwave in our experiment (Figures 5B,D). In terrestrial plants, stress memory has been predicted to last from several days or months (Iqbal and Ashraf, 2007; Rendina González et al., 2018). Together with Jueterbock et al. (2019), our study adds to the emerging knowledge of the length of thermal stress memory in seagrasses which could benefit from future studies to better understand stress memory duration in seagrasses. Also in this context, questions about the inheritance of stress memory in seagrasses deserve future efforts, especially when heat stress can induce and advance flowering in some seagrass species (Diaz-Almela et al., 2007; Blok et al., 2018; Ruiz et al., 2018; Marín-Guirao et al., 2019) as seen in many other plants (Wada and Takeno, 2010; Takeno, 2012, 2016). Heat stress-induced flowering/sexual reproduction can provide an “escaping” mechanism for seagrasses, allowing them to migrate to more favorable areas and/or stabilize the resilience of the plants’ populations by increasing genetic diversity through sexual reproduction (Hughes and Stachowicz, 2004; Procaccini et al., 2007; Ehlers et al., 2008). Further to that, heat stress-induced flowering can also favor transgenerational memory of stress (Molinier et al., 2006; Boyko and Kovalchuk, 2010) in seagrasses, which could potentially secure the existence of threatened species in an era of rapid ocean change (Marín-Guirao et al., 2019).

In addition, the absolute degree and temporal stability of stress-memory demand special attention as priming could play an important role in stabilizing natural populations in the face of more frequent extreme heat events (Oliver et al., 2018; Darmaraki et al., 2019b). In fact, because heat stress often happens chronically in natural conditions, it could contribute to the maintenance of thermal stress memory (Bäurle, 2016) which benefits the resilience of seagrasses. This could partly explain the surprisingly weak effects of repeated heat events on natural populations. After the abrupt *P. oceanica* population decline reported after the 2006 heatwave (Marbà and Duarte, 2010; Jordà et al., 2012) no further mortality has been described after subsequent more intense and longer-lasting heatwaves in the Mediterranean occurred (e.g., 2012, 2015, 2017 see Darmaraki et al., 2019a).

In natural conditions, heat stress often does not occur alone, but in combination with multiple stressors (Gunderson et al., 2016). At this point it is also important to evaluate if heat acclimation and formation of heat-stress memory also prevent damage by other stressors, providing cross-stress memory and tolerance to current and future seagrass threats. Are heat-primed plants more tolerant also to other biotic and abiotic stress?

Controlled lab experiments need to be accompanied by field experiments and field observations after naturally occurring marine heatwaves. Conducting field experiments is often challenging, but new technological advances are promisingly allowing us to conduct more realistic mesocosm experiments and even conduct *in situ* experiments that simulate marine heatwaves (see Egea et al., 2019).

Lastly, although our results suggest the involvement of epigenetic modifications on stress memory in seagrasses, as broadly suggested in terrestrial plants (see reviews from Iwasaki and Paszkowski, 2014; Kinoshita and Seki, 2014; Latzel et al., 2016), the underlying mechanisms are yet to be revealed. Thus, future studies, exploring the mechanisms of stress memory in seagrasses are clearly needed.

## Data Availability Statement

The datasets generated for this study can be found in the all datasets for this study are included in the article/Supplementary Material.

## Author Contributions

HN, LM-G, MP, PR, and GP conceived and designed the experiment. HN, MK performed the experiment. HN analyzed the results. All authors wrote and reviewed the manuscript.

## Conflict of Interest

The authors declare that the research was conducted in the absence of any commercial or financial relationships that could be construed as a potential conflict of interest.

## Footnotes

1 https://www.seatemperature.org/australia-pacific/australia/palm-beach.htm

2 http://115.146.91.129/version3/index.php
