# Supplementary material for: Stress Memory in Seagrasses: First Insight Into the Effects of Thermal Priming and the Role of Epigenetic Modifications
Source: Front Plant Sci. 2020 Apr 28;11:494. doi: 10.3389/fpls.2020.00494 (PMC7199800; doi:10.3389/fpls.2020.00494)
Supplement: Supplementary file 1 [file Data_Sheet_1.PDF]

Stress memory in seagrasses: first insight into the effects of thermal priming and the role of epigenetic modification

Hung Manh Nguyen[1], Mikael Kim[3], Peter Ralph[3], Lázaro Marín-Guirao[1,2†], Mathieu Pernice[3†] and Gabriele Procaccini[1†]

Supplementary data

Selections of best housekeeping genes

Posidonia australis

NormFinder

Gene name	Stability value		Best gene	18S
GADPH	10.623		Stability value	7.817
ef1A	10.750			
18S	7.817		Best combination of two genes	ef1A and 18S
UBI	11.581		Stability value for best combination of two genes	6.718

GeNorm

	Normalisation Factor
GADPH	1.1532
ef1A	0.6867
18S	0.8256
UBI	1.5295

Bestkeeper

BestKeeper vs.	HKG 1	HKG 2	HKG 3	HKG 4
coeff. of corr. [r]	0.934	0.916	0.975	0.855
p-value	0.001	0.001	0.001	0.001

Regression Analysis: HKG vs. BestKeeper				
	GAPDH	ef1A	18S	UBI
	TG 1	TG 2	TG 3	TG 4
	vs.	vs.	vs.	vs.
	BK	BK	BK	BK
coeff. of corr. [r]	0.934	0.916	0.975	0.855
coeff. of det. [r^2]	0.872	0.839	0.951	0.731
intercept [CP]	-0.7048	1.9011	-4.8694	10.0904
slope [CP]	1.2312	0.6345	1.1933	1.0783
SE [CP]	±12.86	±7.576	±7.383	±17.821
p-value	0.001	0.001	0.001	0.001
Power [x-fold]	2.497351726	1.552399636	2.191571	2.07968769

Zostera muelleri

NormFinder

Gene name	Stability value		Best gene	ef1A
Actin	36.326		Stability value	23.198
ef1A	23.198			
GADPH	65.052		Best combination of two genes	ef1A and Tub
Tub	34.808		Stability value for best combination of two genes	25.393

GeNorm

	**Normalisation Factor**
**Actin**	**0.6215**
ef1A	**0.8294**
GADPH	**2.5841**
**Tub**	**0.7508**

Bestkeeper

BestKeeper vs.	HKG 1	**HKG 2**	HKG 3	**HKG 4**
coeff. of corr. [r]	0.908	**0.911**	0.808	**0.912**
p-value	0.001	**0.001**	0.001	**0.001**
Regression Analysis: HKG vs. BestKeeper				
	Actin	**ef1A**	GADPH	**Tub**
	TG 1	**TG 2**	TG 3	**TG 4**
	vs.	**vs.**	vs.	**vs.**
	BK	**BK**	BK	**BK**
coeff. of corr. [r]	0.908	**0.911**	0.808	**0.912**
coeff. of det. [r^2]	0.824	**0.83**	0.653	**0.832**
intercept [CP]	5.6725	**-15.682**	102.903	**-25.2161**
slope [CP]	0.5811	**0.9521**	1.8652	**0.971**
SE [CP]	±16.055	**±25.767**	±81.33	**±26.091**
p-value	0.001	**0.001**	0.001	**0.001**
Power [x-fold]	1.475458754	**1.965988342**	3.67041	**1.9868404**

Summary

Speices	NormFinder (Stab. Value)	GeNorm (Norm. factor)	Bestkeeper [r]
Posidonia australis	**18S** (7.817)	**ef1A** (0.6876)	GAPDH (0.934)
	GAPDH (10.623)	**18S** (0.8256)	**18S** (0.975)
	**ef1A** (10.750)	GAPDH (1.1532)	**ef1A** (0.916)
	UBI (11.518)	UBI (1.5295)	UBI (0.855)
Zostera muelleri	**ef1A** (23.198)	Actin (0.6215)	**Tub** (0.912)
	**Tub** (34.808)	**Tub** (0.7508)	**ef1A** (0.911)
	Actin (36.326)	**ef1A** (0.8294)	Actin (0.908)
	GAPDH (65.052)	GAPDH (2.5841)	GAPDH (0.808)
